# Editorial: Plant-friendly microorganisms as a bio-barrier against pathogens

**DOI:** 10.3389/ffunb.2025.1659453

**Published:** 2025-07-29

**Authors:** Ofir Degani, Maggie Levy, Benjamin A. Horwitz

**Affiliations:** ^1^ Plant Sciences Department, Migal - Galilee Research Institute, Kiryat Shmona, Israel; ^2^ Faculty of Sciences and Technology, Tel-Hai College, Tel Hai, Israel; ^3^ The Robert H. Smith Faculty of Agriculture, Food and Environment, The Hebrew University of Jerusalem, Rehovot, Israel; ^4^ Faculty of Biology, Technion - Israel Institute of Technology, Haifa, Israel

**Keywords:** biological control, crop protection, endophytes, plant disease, host-pathogen interaction, plant microbiome, microbial interactions, microorganism communities

Plants are exposed to a wide range of pathogenic species that coexist within complex microbial communities (Jones et al., 2019). The combinations of pathogens in these communities can exacerbate plant diseases. Alternatively, they may inhibit one another through antagonistic interactions. According to the classical disease triangle in phytopathology, disease development requires the interaction of a susceptible host, a virulent pathogen, and an environment conducive to disease progression. The plant microbiome plays a critical role in modulating both host resistance and the local environment in the plant host.

Microbial communities residing in seeds, the rhizosphere (the soil region adjacent to plant roots), and the phyllosphere (the plant’s aerial parts) consist of opportunistic pathogens and non-pathogenic organisms. These microbes, including diverse bacteria and fungi, may either cooperate or compete for plant-derived resources within shared ecological niches (Berg et al., 2020). Importantly, some of these endophytes and other microbiome constituents can suppress pathogens, thus promoting plant health (Degani et al.; Srivastava et al., 2025). Understanding the structure and function of these microbial communities under varying biotic and abiotic stress conditions offers promising new avenues for biological disease control.

Indeed, agriculture faces increasing pressure to reduce reliance on chemical pesticides while maintaining crop productivity under rising biotic and abiotic stress conditions. Beneficial microorganisms, including plant growth-promoting rhizobacteria such as *Bacillus velezensis* (Wockenfuss et al.) and fungi such as *Trichoderma* spp. (Akanksha et al.) and the entomopathogenic fungus *Metarhizium* (Mesquita et al.), offer a promising alternative by serving as a first line of defense, forming a dynamic and responsive bio-barrier that suppresses pathogens and enhances plant resilience (Mendes et al., 2011; Srivastava et al., 2025).

In this Research Topic, Akanksha et al. optimized the production and characterization of chitinase enzymes from *Trichoderma* spp. and demonstrated their strong antifungal activity against soil-borne pathogens affecting apple nurseries, with *T. atroviride* UHFTA005 showing the highest *in vitro* and *in vivo* disease suppression. These findings highlight the potential of Trichoderma-derived chitinases as effective biocontrol agents in the framework of managing apple root diseases in Himachal Pradesh, India. Likewise, Wockenfuss et al. showed that *Bacillus velezensis*, isolated from agricultural soil, exhibits strong *in vitro* antifungal activity against several plant pathogenic fungi and an oomycete. The bacterium alters fungal development, disrupting normal hyphal growth and appressoria formation, suggesting its potential as a broad-spectrum biocontrol agent. Even more so, pathogens’ interactions can affect disease severity, as demonstrated by Degani et al. The research explored interactions between *Magnaporthiopsis maydis* and newly identified endophytic fungi isolated from sweet corn seeds. Several isolates, including *Fusarium* sp. and *Aspergillus* species, demonstrated antagonistic activity against *M. maydis*, suggesting that native seed microflora may serve as a basis for novel biocontrol strategies. The benefits of root symbionts can extend to controlling parasitic insects as well. Mesquita et al. reviewed the potential of the entomopathogenic fungus *Metarhizium*, which plays a key role in Brazilian sugarcane agriculture due to its insecticidal properties and activity as a plant growth-promoting symbiont.

Microbial communities contribute to plant disease suppression through competitive exclusion, production of antagonistic metabolites, and modulation of host immune responses. Numerous studies have demonstrated that pathogen attack can trigger plants to recruit beneficial microbes via root exudates (Rolfe et al., 2019; Chepsergon and Moleleki, 2023). The mechanisms underlying this microbe-mediated protection include antibiosis, whereby beneficial microbes produce antimicrobial compounds that inhibit pathogen growth; niche competition, in which early colonizers efficiently utilize nutrients and occupy ecological niches, thereby excluding pathogens (Degani et al.; Srivastava et al., 2025). Furthermore, beneficial microbes can induce systemic resistance, mainly through the jasmonic acid and ethylene signaling pathways, priming, in this way, the plant’s defensive capacity against a broad range of pathogens (Compant et al., 2025). Key taxa studied in this Research Topic and related works include: *Bacillus* spp. and *Pseudomonas fluorescens* with robust colonization and bioactive metabolite production (Degani et al.; Wockenfuss et al.) (Mendes et al., 2011; Rabbee et al., 2023). Additionally, *Trichoderma* spp. and arbuscular mycorrhizal fungi have dual roles in pathogen suppression and abiotic stress mitigation (Akanksha et al.) (Jin and Alberti, 2025). Implementing microbial bio-barriers offers several benefits, including reduced dependency on synthetic fungicides, enhanced plant growth, nutrient uptake, stress resilience, and compatibility with integrated pest management and organic practices (Vishwakarma et al., 2020). Lab and field trials indicate that consortia of beneficial microbes significantly improve disease control efficacy when adapted to local soil and crop conditions compared to single-strain inoculants (Jones et al., 2019; Rolfe et al., 2019; Berg et al., 2020). The articles in this Research Topic illustrate plant-associated beneficial microbes’ ecological, biochemical, and practical roles in suppressing pathogens and enhancing crop health. Their multifunctionality offers an avenue for environmentally sound disease control strategies. Continued research is essential to optimize their use in diverse agroecosystems and contribute to the global transition toward sustainable agriculture.

Despite substantial progress, key research gaps remain: first, the inconsistency in the performance or effectiveness of a biocontrol treatment under real-world field conditions should be assessed. Many strains show promise *in vitro* or greenhouses but lose effectiveness under field conditions due to complex soil–plant–microbe interactions (Fadiji and Babalola, 2020). Second, understanding host–microbe recognition and stable root/endosphere colonization is essential for consistent performance (Wallace and May, 2018). Omics-based tools (e.g., metagenomics, metabolomics) should be employed to identify beneficial strains naturally antagonistic to pathogens, unravel their modes of action, and optimize synthetic microbial communities that function synergistically in the field. In this sense, nano-/formulation technologies that ensure stable, targeted delivery of bio-pesticides or their metabolites are essential. Finally, developing stable, scalable, and shelf-stable microbial products remains a critical challenge. We should ensure host specificity and safety, reducing risks of non-target effects or pathogen gene transfer.
